# Promoting genotype-independent plant transformation by manipulating developmental regulatory genes and/or using nanoparticles

**DOI:** 10.1007/s00299-023-03037-2

**Published:** 2023-06-14

**Authors:** Tingwei Yan, Quancan Hou, Xun Wei, Yuchen Qi, Aqing Pu, Suowei Wu, Xueli An, Xiangyuan Wan

**Affiliations:** 1 Research Institute of Biology and Agriculture, Shunde Innovation School, School of Chemistry and Biological Engineering, University of Science and Technology Beijing, Beijing, 100083 China; 2 Zhongzhi International Institute of Agricultural Biosciences, Beijing, 100083 China; 3 Beijing Engineering Laboratory of Main Crop Bio-Tech Breeding, Beijing International Science and Technology Cooperation Base of Bio-Tech Breeding, Beijing Solidwill Sci-Tech Co. Ltd., Beijing, 100192 China

**Keywords:** Developmental regulatory genes, Genotype-independent transformation, Nanoparticles, Root organogenesis, Shoot organogenesis, Somatic embryogenesis

## Abstract

**Key message:**

This review summarizes the molecular basis and emerging applications of developmental regulatory genes and nanoparticles in plant transformation and discusses strategies to overcome the obstacles of genotype dependency in plant transformation.

**Abstract:**

Plant transformation is an important tool for plant research and biotechnology-based crop breeding. However, Plant transformation and regeneration are highly dependent on species and genotype. Plant regeneration is a process of generating a complete individual plant from a single somatic cell, which involves somatic embryogenesis, root and shoot organogeneses. Over the past 40 years, significant advances have been made in understanding molecular mechanisms of embryogenesis and organogenesis, revealing many developmental regulatory genes critical for plant regeneration. Recent studies showed that manipulating some developmental regulatory genes promotes the genotype-independent transformation of several plant species. Besides, nanoparticles penetrate plant cell wall without external forces and protect cargoes from degradation, making them promising materials for exogenous biomolecule delivery. In addition, manipulation of developmental regulatory genes or application of nanoparticles could also bypass the tissue culture process, paving the way for efficient plant transformation. Applications of developmental regulatory genes and nanoparticles are emerging in the genetic transformation of different plant species. In this article, we review the molecular basis and applications of developmental regulatory genes and nanoparticles in plant transformation and discuss how to further promote genotype-independent plant transformation.

## Introduction

Plant transformation is a method that delivers foreign DNA into regeneration-competent cells through the *Agrobacterium*-mediated method, biolistic (also called particle bombardment), pollen tube transformation, electroporation and so on. Among them, *Agrobacterium*-mediated and biolistic methods are the most common plant transformation methods (An et al. 2019, 2020; Zhang et al. 2018, 2021b; Zhu et al. 2020). Regenerative cells can derive from the proliferation of undifferentiated meristem cells of explants and then develop into intact plants through direct organogenesis. However, in many plants, regenerative cells are derived from the reprogramming of differentiated somatic cells and regain the ability for proliferation competence through dedifferentiation. Regenerative cells derived in this way develop into intact plants by de novo organogenesis and somatic embryogenesis (Feher 2019; Gaillochet and Lohmann 2015; Ikeuchi et al. 2016; Steward et al. 1958; Sugimoto et al. 2011; Xu and Hu 2020). Direct organogenesis regenerates adventitious shoots or roots directly from explants, while indirect organogenesis requires induction of pluripotent non-embryonic callus on the callus-inducing medium (CIM), and the callus then develops into adventitious shoots or roots. The callus is a highly heterogeneous group of cells, and its organized structure resembles lateral root primordia (Atta et al. 2009). Somatic embryo regeneration, namely somatic embryogenesis, depends on the totipotency of plant cells (Feher 2019). Somatic embryogenesis can induce somatic embryos directly without an intermediate embryonic callus or indirectly following an embryonic callus stage. In plant research, both plant genetic engineering and genome editing technologies that promote functional genomic research and accelerate crop trait improvement greatly depend on plant transformation (Altpeter et al. 2016; Fang et al. 2022; Hou et al. 2022a; Jiang et al. 2021; Liu et al. 2022a, b; Wei et al. 2022, 2023). However, plant transformation and regeneration rely highly on species and genotype, which are major limiting factors for developing and applying genetic engineering and genome editing technologies.

Since various factors influence callus formation and regeneration, successful plant transformation has to optimize several external factors such as explant types, pH, and basal media composition. Considering that most regeneration initiates from the cut place, the wound stress may be a trigger of plant regeneration (Ikeuchi et al. 2013). Recent studies showed that hormones and developmental regulatory genes play critical roles in callus induction and plant regeneration. Exogenous hormones induce callus formation in aerial explants with the elimination of leaf identity (He et al. 2012; Lee and Seo 2018). Then, developmental regulatory genes regulate de novo shoot and root regeneration in root and aerial explants (Kareem et al. 2015; Liu et al. 2014). In aerial explant-initiated plant regeneration, the elimination of leaf identity is primarily achieved through epigenetic regulation (He et al. 2012; Lee and Seo 2018). Overall, wounds, hormones, developmental regulatory genes, and epigenetic modifications are essential factors for plant regeneration. Recent studies showed the successful transformation of recalcitrant species through manipulating developmental regulatory genes (Aregawi et al. 2022; Hoerster et al. 2020; Lowe et al. 2018). Alternatively, nanoparticles (NPs) could penetrate the plant cell wall without external force and can be broadly applied to different plant species. In addition, nanomaterials (NMs) can protect cargoes from degradation and reach previously inaccessible plant tissues, cellular and subcellular locations. All these properties make NPs promising materials for exogenous biomolecule delivery and several recent studies showed the successful usage of NPs to deliver genes into plant cells for genetic engineering and genome editing (Demirer et al. 2019a; Kwak et al. 2019; Wang et al. 2020).

In this review, we performed a bibliometric analysis of 2414 publications selected by the related searches of plant transformation methods to gain a brief overview of the research history and status. We summarize and discuss the molecular basis of wounds, hormones, developmental regulatory genes, and epigenetic modifications in plant regeneration and the application of developmental regulatory genes in plant transformation. In addition, we also summarize the uptake and translocation of recently emerged NPs in plant cells and their application in plant transformation. Finally, we discuss the potential for genotype-independent plant transformation based on these advances.

## A brief overview of plant transformation methods

**Fig. 1:**
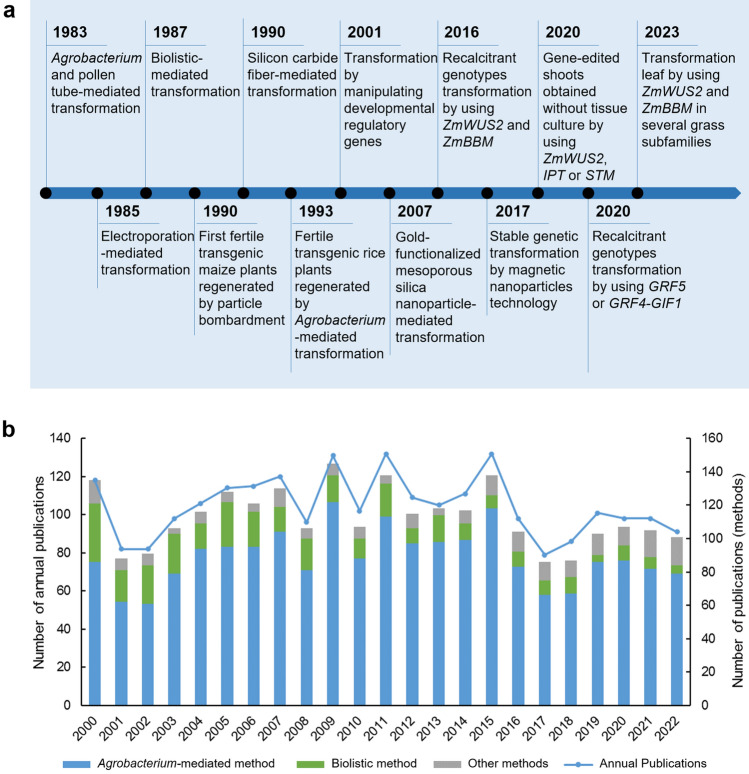
Landmark events and related publications of plant transformation. **a** Timeline of landmark events in plant transformation. **b** Publications related to plant transformation from 2000 to 2022. Publications on seven plant transformation methods, including the *Agrobacterium*-mediated method, biolistic, pollen tube-mediated method, electroporation, silicon carbide fiber-mediated method, polyethylene glycol-mediated method, and nanoparticles delivery

The mechanism of *Agrobacterium*-mediated plant transformation is the transfer of foreign genes carried between the Ti plasmid T-DNA boundaries to the plant cell nucleus and subsequent transient transgene expression or integration into the plant genome. In 1983, with the successful regeneration of the transgenic *Nicotiana tabacum* transformed using the *Agrobacterium*-mediated method, the “starting line” was drawn for plant transformation (Fraley et al. 1983; Herrera-Estrella et al. 1983). Meanwhile, a study reported that a pollen tube-mediated method could successfully transform *Gossypium hirsutum* (Zhou et al. 1983). From then, leaf discs of a wide range of dicotyledon plants were successfully infected by *Agrobacterium* (Horsch et al. 1985). However, monocots, particularly the graminaceous crops, cannot be infected for a long time via this method. Other methods, such as biolistic, electroporation and silicon carbide fiber-mediated methods, are developed and applied to monocot transformation (Fromm et al. 1985; Kaeppler et al. 1990; Klein et al. 1987). In 1990, the first fertile transgenic *Zea mays* plants transformed by the biolistic method were regenerated (Gordon-Kamm et al. 1990). This method can deliver biomolecules to a broader range of plant species, but plant tissue is often damaged under high bombardment pressures. In addition, biolistic technology requires specialized equipment, which limits its wide application. It is necessary to develop *Agrobacterium* infecting method for monocots because the *Agrobacterium*-mediated method is easy to perform, low cost, and shows higher transformation efficiency than other methods. Several studies found that monocots can not produce enough inducers, such as phenolic compounds, at injury sites (Stachel et al. 1985). This could be the reason for the recalcitrance to *Agrobacterium* infection as these compounds are required for the activation of virulence (Vir) genes on the Ti plasmid, which is needed for inducing plant tumor production (Stachel et al. 1985). Indeed, transgenic *Oryza sativa* is obtained through *Agrobacterium*-mediated infection of immature embryos with the addition of phenolic compounds (Chan et al. 1993). These landmark events in plant transformation are illustrated in Fig. 1A. These methods were widely used in plant transformation from 2000 to 2022, among which the *Agrobacterium*-mediated method is the most used, followed by the biolistic method (Fig. 1B). However, the bottleneck of plant transformation and regeneration is species and genotype dependence.

Several studies have recently attempted to transform recalcitrant plant species and genotypes. For example, a method called *in planta* particle bombardment (iPB) is developed for *Triticum aestivum* transformation (Hamada et al. 2018). The iPB method is used to deliver Cas9/gRNA plasmids to shoot apical meristem (SAM) of imbibed seeds and regenerated genome-edited plants (Hamada et al. 2018). This method makes it possible to transform other recalcitrant plant species. *Agrobacterium*-mediated *Vigna unguiculata* embryonic axis transformation achieved transformation frequencies between 4 and 37% in many genotypes (Che et al. 2021). In addition, SAM cells were used as explants to transform recalcitrant *G. hirsutum* genotypes and successfully obtained transgenic plants (Ge et al. 2023). However, few studies showed the use of SAM or embryonic axis as explants to promote genotype-independent transformation. Another strategy for transforming recalcitrant species and genotypes is manipulating developmental regulatory genes and this strategy is widely used in genotype-independent plant transformation (Aregawi et al. 2022; Hoerster et al. 2020; Lowe et al. 2018). Recently, several plant species have achieved stable genetic transformation via magnetic nanoparticles (MNPs) technology (Wang et al. 2022b; Zhao et al. 2017). In the following sections, we will discuss in detail the mechanism and application of developmental regulatory genes and NPs to promote genotype-independent plant transformation.

## Molecular basis of somatic embryogenesis, root and shoot organogeneses

Intact plant regeneration from a single somatic cell has to experience somatic embryogenesis, root and shoot organogeneses, and these processes require proper in vitro conditions and involve complicated in vivo signaling and transcriptional networks triggered or regulated by wounds, hormones, developmental regulatory genes and epigenetic reprogramming. Factors affecting somatic embryogenesis, root and shoot organogeneses and their molecular basis are summarized in the succeeding texts.

### Molecular basis of somatic embryogenesis

**Fig. 2:**
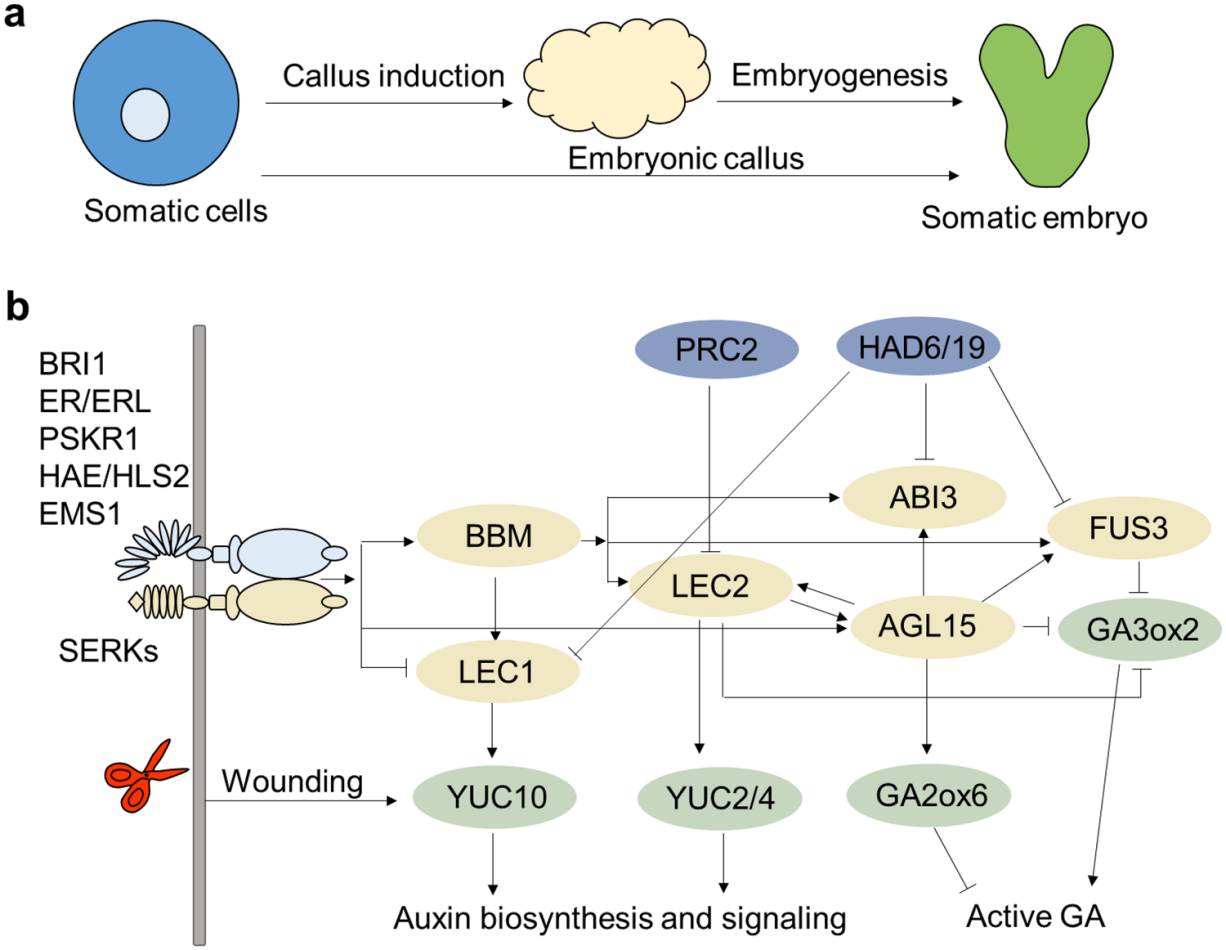
Schematic representation and molecular regulatory network of somatic embryogenesis. **a** Schematic representation of somatic embryogenesis. Somatic embryos can be induced through somatic embryogenesis directly or indirectly. In indirect induction, embryonic callus is induced from plant somatic cells on the callus-inducing medium (CIM), and then somatic embryo formation. **b** Molecular regulatory network of somatic embryogenesis. Developmental regulatory genes are in yellow; hormone biosynthesis- and signaling-related genes are in green and epigenetic modification-related genes are in blue. Arrows and bar-head arrows represent activation and repression, respectively (color figure online)

Somatic embryogenesis occurs in many plant species when they are incubated on an auxin-containing medium and then transferred to an auxin-free medium (Ikeda-Iwai et al. 2002; Lu et al. 1983; Wernicke and Brettell 1980). During indirect somatic embryogenesis, embryonic callus formation is first activated on an auxin-rich medium (Ikeda-Iwai et al. 2002). The subsequent absence of auxin in the medium leads to the de novo establishment of auxin gradients in the embryonic callus (Fig. 2A). The gradient auxin distribution initiates a developmental program similar to zygotic embryogenesis, possibly activating the auxin transporter PIN-PORMED1 (PIN1) polar localization (Liu et al. 1993; Su et al. 2009). *WUSCHEL* (*WUS*), which determines stem cell fate in SAM, is induced by the established auxin gradient and polar auxin transport, and promotes somatic embryogenesis (Su et al. 2009).

Since most regeneration occurs at wounded loci, wound stress has long been considered a trigger for plant regeneration (Ikeuchi et al. 2013). Wound stress is perceived via damage-associated molecular modules, including cell wall-derived oligogalacturonic acid (Bishop et al. 1981) and extracellular adenosine triphosphate (ATP) (Choi et al. 2014; Tanaka et al. 2014). The ATP is released as a danger signal during plant damage, inducing cytoplasmic calcium signaling and a burst of reactive oxygen species (Choi et al. 2014; Tanaka et al. 2014). The local wound signals are further translated into electrical signals, such as cation channel GLUTAMATE RECEPTOR-LIKEs, which are transmitted to other parts of the plant to induce epigenetic modifications, transcriptional changes and phytohormone synthesis (Ikeuchi et al. 2017; Mousavi et al. 2013).

Somatic embryogenesis receptor-like kinase1 (SERK1) is a Leu-rich repeat (LRR) transmembrane receptor-like kinase (PLK) that might co-regulate the plant differentiation process with other specific receptor-like kinases. Ectopic expression of *SERK1* has been taken as a strategy for improving the somatic embryogenesis efficiency of *Coffea canephora* (Perez-Pascual et al. 2018)*, Arabidopsis thaliana* (Hecht et al. 2001)*,* and *O. sativa* (Hu et al. 2005). SERK1 regulates somatic embryogenesis by activation of auxin biosynthesis, auxin transport, and probably also auxin perception, leading to the expression of early-stage homeotic genes, including *WUS*, the AP2/ERF transcription factor *Baby boom* (*BBM*) and the MADS-box transcription factor *Agamous-like15* (*AGL15*), and the repression of late-stage homeotic genes such as *Leafy cotyledon1* (*LEC1*) (Perez-Pascual et al. 2018). The BBM, LEC1, LEC2, and AGL15 transcription factors play essential roles in early embryogenesis. *LEC1* and *LEC2*, as well as two other transcription factors,* Abscisic acid insensitive3* (*ABI3*) and *FUSCA3* (*FUS3*) are up-regulated by BBM in somatic embryogenesis (Horstman et al. 2017). In addition, LEC2 rapidly activates the expression of *AGL15* (Braybrook et al. 2006). Interestingly, *LEC2*, *FUS3*, and *ABI3* were identified as direct target genes of AGL15 (Zheng et al. 2009). These data suggest that feedback regulation exists in gene regulatory networks during embryogenesis. In addition, *LEC2* is a mediator of auxin biosynthesis and signaling. LEC2 induces *YUCCA2* (*YUC2*) and *YUC4* (Stone et al. 2008), which encode auxin biosynthesis enzymes, while LEC1 activates the *YUC10* (Junker et al. 2012). AGL15 directly upregulates *GA2ox6*, a GA catabolic enzyme, and represses the GA biosynthesis gene *GA3ox2* leading to a reduction of biologically active GA in *Arabidopsis* (Wang et al. 2004; Zheng et al. 2009). *Ga3ox2* is also repressed by LEC2 and FUS3 and is ectopically activated in the loss-of-function mutants of *lec2* and *fus3* (Curaba et al. 2004).

Epigenetic reprogramming occurs in many plant developmental processes and regeneration (Hou and Wan 2021; Hou et al. 2022b). Studies have shown that epigenetic modifications, including histone modifications and DNA methylation, suppress regenerative potential and maintain the differentiated status of plant cells (Chen and Dent 2014; Ikeuchi et al. 2015a; Lee and Seo 2018). A chromatin regulator POLYCOMB REPRESSIVE COMPLEX 2 (PRC2) promotes trimethylation on lysine 27 of histone H3 (H3K27me3) to represses gene expression (Holec and Berger 2012). In *Arabidopsis*, loss-of-function mutants in PRC2 complex develop normal root hairs but fail to maintain the differentiated state and generate callus and somatic embryos (Ikeuchi et al. 2015b). The *Wound-induced dedifferentiation 3* (*WIND3*) and *LEC2* are target genes of PRC2, and ectopic overexpression of *WIND3* and *LEC2* partly phenocopies the *prc2* mutants (Ikeuchi et al. 2015b). These findings suggested that PRC2-mediated gene repression is essential for maintaining the differentiated cell state. Histone acetylation is a permissive histone mark and plays an essential role in somatic embryogenesis (Kadosh and Struhl 1998; Rundlett et al. 1998). *Arabidopsis* plants treated with trichostatin A (TSA), an inhibitor of histone deacetylases (HDAC), resulted in growth arrest and enhanced transcription of *LEC1, FUS3*, and *ABI3* during germination (Tanaka et al. 2008). In addition, an *HAD6/HAD19* double-repression line generated embryo-like structures on the true leaves. These phenotypes of the repression line can be rescued by *lec1* (Tanaka et al. 2008). Thus, HDA6 and HDA19 redundantly regulate the inhibition of embryonic properties by repressing embryo-specific genes during germination in *Arabidopsis*. In *Brassica napus*, repressing histone deacetylase activity with TSA resulted in a significant increase in cell transition from pollen to embryogenic growth in male gametophytes (Li et al. 2014). Interestingly, TSA with heat treatment greatly increased the formation of somatic embryos (Li et al. 2014). Thus, heat stress and histone deacetylation may synergistically regulate somatic embryogenesis (Fig. 2B).

### Molecular basis of de novo root organogenesis

**Fig. 3:**
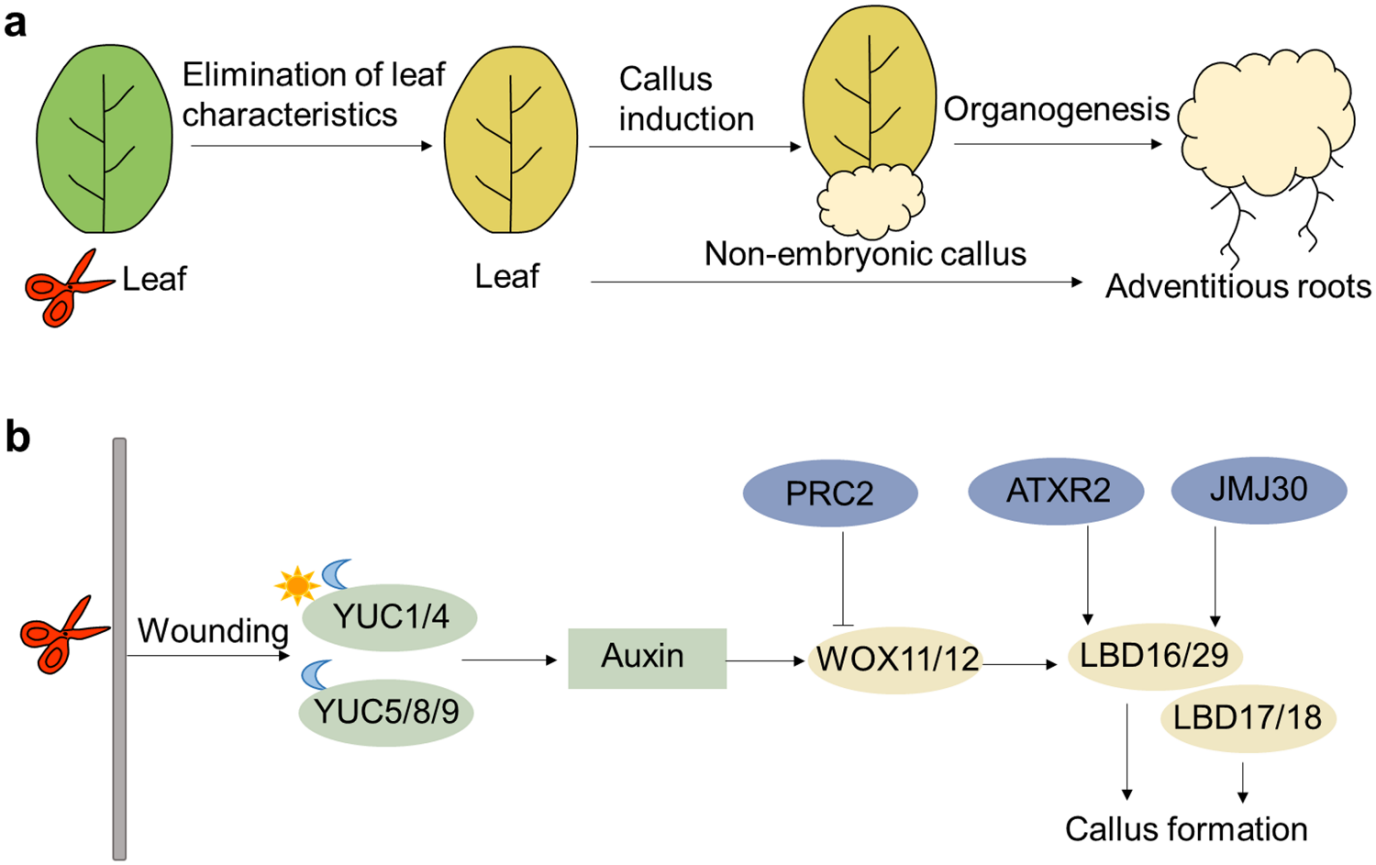
Schematic representation and molecular regulatory network of de novo root organogenesis. **a** Schematic representation of de novo root organogenesis. First, leaf explants have to eliminate leaf characteristics. Then, adventitious roots are induced directly or indirectly through de novo root organogenesis. In indirect induction, the non-embryonic callus is induced on the callus-inducing medium (CIM), and these cells then develop into adventitious roots on the root-inducing medium (RIM). **b** Molecular regulatory network of de novo root organogenesis. Developmental regulatory genes are in yellow; hormone biosynthesis- and signaling-related genes are in green and epigenetic modification-related genes are in blue. Symbols of the sun and the moon represent light and dark conditions, respectively. Arrows and bar-head arrows represent activation and repression, respectively (color figure online)

The pericycle cells between the endodermis and stele have the potential to generate new lateral roots (Beeckman and De Smet 2014). *Arabidopsis* explants incubation on CIM and the root-inducing medium (RIM) strongly promote root regeneration from pericycle cells. Culturing hypocotyl explants on RIM after pretreatment on CIM induces a large number of roots, whereas only a few roots form when they are inoculated on RIM without the pretreatment. In contrast to hypocotyl explants, root explants with lateral root meristem primordia efficiently promote root formation when directly cultured on RIM. These results suggest that CIM induces the pluripotent non-embryonic callus generation and these cells then further develop into adventitious roots on RIM (Fig. 3A).

Several studies have shown that wounds, hormones, developmental regulatory genes, and epigenetic modifications affect de novo root organogenesis. Under either light or dark conditions, *YUC1* and *YUC4* are rapidly activated in response to wounding, promoting auxin biogenesis in mesophyll and competent cells, while *YUC5*, *YUC8*, and *YUC9* mainly respond to dark conditions. Overall, *YUC* genes enhanced the auxin level in leaf explants during de novo root organogenesis (Chen et al. 2016). Wuschel related homeobox11 (WOX11), a homeobox gene, responds to wounding-induced auxin signaling together with its homolog WOX12 to upregulate *Lateral organ boundaries domain 16* (*LBD16*) and *LBD29*, resulting in the fate transition from leaf procambium or parenchyma cells to root founder cells (Liu et al. 2014). Notably, the auxin response elements (AuxREs) in the promotor of *WOX11* are essential for its induction in leaf explants, indicating that the auxin signaling pathway directly activates *WOX11* expression during root regeneration (Liu et al. 2014). Thus, this novel regulatory mechanism links wounding and hormonal signaling to organ formation during regeneration. The other two *LBD* genes, *LBD17* and *LBD18* are also rapidly and significantly induced by CIM. In *Arabidopsis*, ectopic expression of each of the four *LBD* genes is sufficient for spontaneous callus formation in the absence of exogenous phytohormones, and inhibition of *LBD* function suppresses CIM-induced callus formation (Fan et al. 2012). These results support that *LBD* transcription factors play essential roles during the callus induction process. Collectively, these regulatory pathways together promote the auxin-mediated establishment of root meristems.

Epigenetic regulation, such as PRC2-mediated repression, regulates *WOX11* to influence plant cell fate transition (Ikeuchi et al. 2013; Liu et al. 2014). In addition, JUMONJI C domain-containing protein 30 (JMJ30) binds to the promoters of *LBD16* and *LBD29* with Auxin response factor 7/19 (ARF7/ARF19) which are transcriptional activators of early auxin response, removes the methyl groups from H3K9me3, and promotes *LBD* expression (Lee et al. 2018). Arabidopsis trithorax-related 2 (ATXR2) is recruited to *LBD16* and *LBD29* promoters through ARF-JMJ30 complex and promotes trimethylation on lysine 36 of histone H3 (H3K36me3) to further promotes *LBD* expression during callus formation (Lee et al. 2017). A schematic gene regulatory network during root organogenesis is illustrated in Fig. 3B.

### Molecular basis of de novo shoot organogenesis

**Fig. 4:**
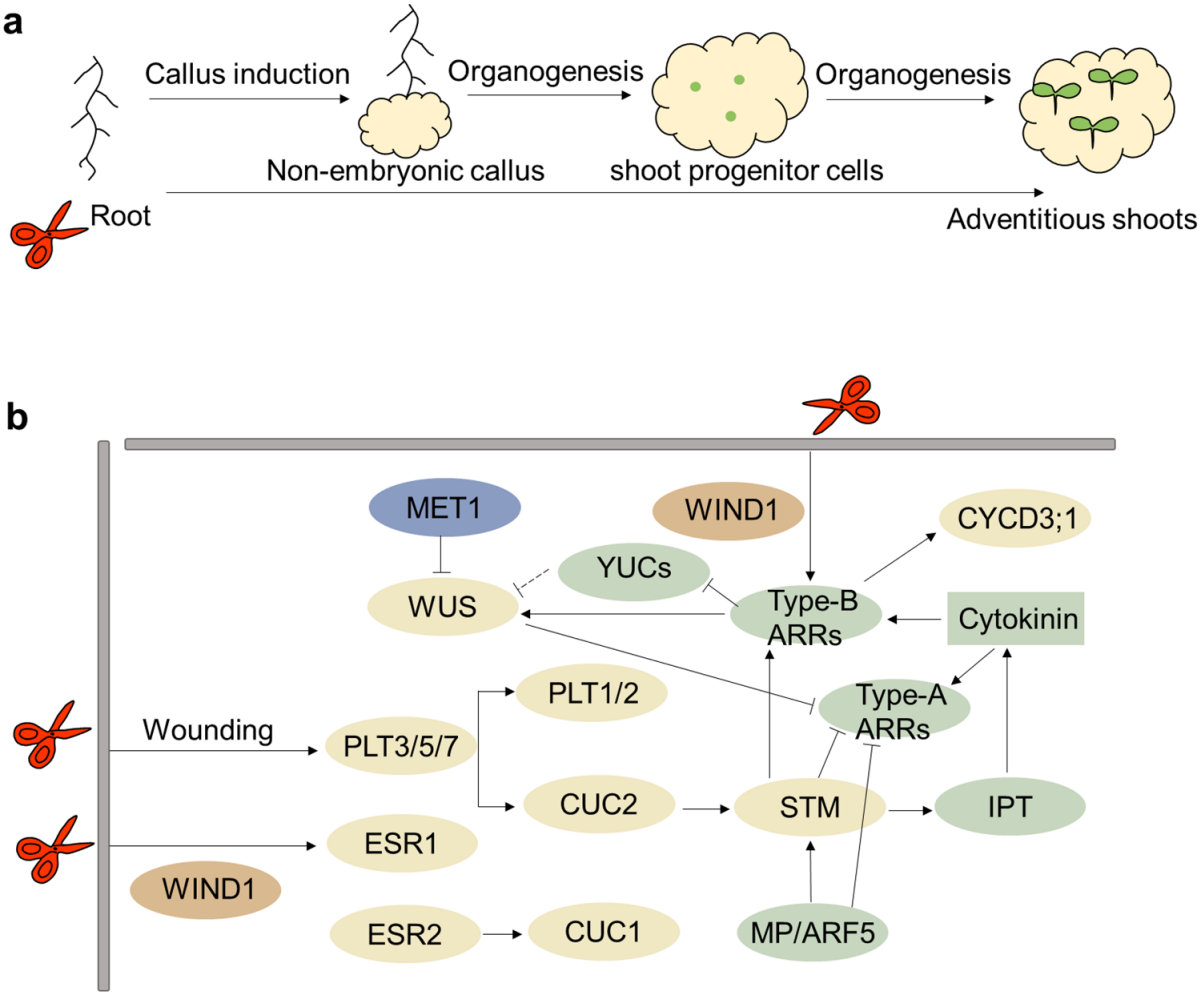
Schematic representation and molecular regulatory network of de novo shoot organogenesis. **a** Schematic representation of de novo shoot organogenesis. Adventitious shoots are directly or indirectly induced by de novo shoot organogenesis. In indirect induction, the non-embryonic callus is induced on the callus-inducing medium (CIM), and the callus then develops into adventitious shoots through two developmental processes: shoot progenitor cell regeneration and shoot formation after transferring to the shoot-inducing medium (SIM). **b** Molecular regulatory network of de novo shoot organogenesis. Wound-related genes are in brown; developmental regulatory genes are in yellow; hormone biosynthesis- and signaling-related genes are in green and epigenetic modification-related genes are in blue. Arrows and bar-head arrows represent activation and repression, respectively (color figure online)

Like many other plants, *Arabidopsis* explants do not readily regenerate shoots. However, culturing *Arabidopsis* explants on CIM and the shoot-inducing medium (SIM) rich in cytokinin strongly promotes shoot regeneration from pericycle cells (Atta et al. 2009; Che et al. 2007; Valvekens et al. 1988). The CIM-induced callus possesses root meristem characteristics. Thus, it is easy to regenerate roots when the CIM-induced callus is transferred to RIM. Establishing the identity of root meristem and further root development can be regulated through auxin-induced transcriptional cascade (Ozawa et al. 1998). In contrast, shoot regeneration may be more complex because it requires the transition from root meristem fate to shoot meristem fate. CIM induces pluripotent non-embryonic callus, which develops into adventitious shoots through two developmental processes: shoot progenitor cell regeneration and shoot formation after transferring to SIM (Kareem et al. 2015) (Fig. 4A).

De novo shoot organogenesis is also regulated by wounds, hormones, developmental regulatory genes, and epigenetic modifications. Studies showed an AP2/ERF transcription factor, *WIND1* and its close homologs *WIND2*, *WIND3*, and *WIND4* are rapidly induced by wounding, and these genes promote cell dedifferentiation and subsequent callus formation in *Arabidopsis* (Iwase et al. 2011a, b). WIND1 upregulates *enhancer of shoot regeneration1/dornröschen* (*ESR1/DRN*), encoding another AP2/ERF transcription factor, and promotes shoot regeneration in *Arabidopsis* (Banno et al. 2001; Iwase et al. 2017). In addition, *WIND1* induces the B-type *Arabidopsis response regulators* (*ARRs*)-mediated cytokinin response (Iwase et al. 2011a). Double mutants of type-B *ARRs* (*arr1-3arr12-1* and *arr1-3arr10-5*) display reduced callus formation. The expression of core cell cycle regulator *Cyclin D3* (*CYCD3;1*) is downregulated in the *arr1-3arr12-1* double mutant and triple loss-of-function mutants of *cycd3;1–3* have low callus formation efficiency (Ikeuchi et al. 2017). Thus, these results suggest that wounding induces cytokinin signaling and then promotes cell cycle activation at wounded sites. Other AP2/ERF transcription factors, PLETHORA3 (PLT3), PLT5, and PLT7, are also induced after wounding, and *plt357* triple mutants are less effective in callus formation (Ikeuchi et al. 2017). In addition, overexpression of *PLT5* successfully obtained transgenic plants of *Antirrhinum majus* and *Brassica rapa* (Lian et al. 2022).

Induction of *PLT3, PLT5*, and *PLT7* are among the earliest transcriptional responses induced by CIM and then the essential root meristem regulators *PLT1* and *PLT2* are activated (Aida et al. 2004; Galinha et al. 2007; Kareem et al. 2015). In addition, *Cup-shaped cotyledon2* (*CUC2*), encoding a NAC family transcription factor, is also activated by PLT3, PLT5, and PLT7. Several pieces of evidence showed that CUC proteins are critical for shoot formation in the callus (Kareem et al. 2015). Overexpression of *CUC1* or *CUC2* enhances the adventitious shoot formation of calli derived from *Arabidopsis* hypocotyls (Daimon et al. 2003). Other transcription factors, such as ESR2, enhance shoot regeneration by directly regulating *CUC1* transcription (Ikeda et al. 2006). After transferring to SIM, the essential shoot stem cell regulator WUS is induced (Gordon et al. 2007). The *CUC2*-expressing cells continue to proliferate and form promeristems in which PIN1 and a homeodomain transcription factor shoot meristemless (STM) are upregulated and further promote the formation of functional shoot meristems (Gordon et al. 2007). Overexpression of *BnSTM* induces type-B *ARRs* and represses type-A *ARRs* (Elhiti and Stasolla 2012). Activation of STM using an inducible system resulted in a rapid and dramatic increase of *isopentenyl transferase 7* (*IPT7*), encoding a cytokinin biosynthesis gene (Yanai et al. 2005). In addition, *MPΔ*, an irrepressible variant of *Monopteros* (*MP*)/*ARF5*, promotes de novo shoot formation by activating the expression of *STM* and repressing the expression of *ARRs-A* (Ckurshumova et al. 2014; Krogan et al. 2012; Zhao et al. 2010).

*WUS* is essential for the maintenance of the stem cell niche in SAMs (Laux et al. 1996). Recent studies revealed that type-B ARRs activate the transcription of *WUS*. Type-B ARRs also inhibit auxin accumulation by repressing *YUCs* and indirectly inducing the expression of *WUS* (Meng et al. 2017). In addition, WUS directly represses the transcription of *type-A ARRs* (Leibfried et al. 2005). Like many other regeneration regulators, epigenetic marks modulate *WUS* expression during shoot regeneration (Li et al. 2011). Loss-of-function of a DNA methyltransferase 1 (MET1) led to increased *WUS* expression and accelerated developmental speed of in vitro shoot regeneration (Li et al. 2011) (Fig. 4B).

## Application of developmental regulatory genes for genotype-independent plant transformation

Developmental regulatory genes that promote plant regeneration have been used to improve transformation efficiency and promote genotype-independent plant transformation. Ectopic expression of *LEC1*, *L1L,* or *LEC2* in *Arabidopsis* (Lotan et al. 1998)*, Picea abies* (Uddenberg et al. 2016)*, Citrus sinensis* (Zhu et al. 2014) and *Theobroma cacao* (Shires et al. 2017) promotes embryo-like structure and somatic embryo formation but cannot obtain regenerated transgenic plants. However, inducible expression of *LEC2* by β-estradiol could regenerate transgenic plants, though the regenerated plant displayed abnormal phenotypes (Rashid et al. 2007). AGL15 promotes the generation of secondary embryos from zygotic embryos and these secondary embryos maintain the potential for embryogenic development (Harding et al. 2003). Ectopic expression of *AGL15* also enhances somatic embryo formation from the shoot apical meristem (Harding et al. 2003). Overexpression of *GmAGL15,* an ortholog of *Arabidopsis AGL15,* promotes somatic embryo development in *Glycine max* (Thakare et al. 2008). In *G. hirsutum*, overexpression of either *GhAGL15-1*, *GhAGL15-3*, or *GhAGL15-4* promotes the embryogenic potential of transgenic calli (Yang et al. 2014).

Overexpression of *TaWOX5* increases the transformation efficiency of multiple *T. aestivum* varieties without genotype dependency (Wang et al. 2022a). Ectopic expression of *AtWOX2/8/9* led to a range of abnormal phenotypes in tobacco (Kyo et al. 2018). Overexpression of *Z. mays* homeobox gene *knotted1* (*Zmkn1*) obtains a large number of transgenic calli and shoots on a hormone-free medium without antibiotic selection in tobacco. Under the same conditions, no callus or shoot was generated from explants that were infected with an *Agrobacterium* strain harboring the *NPTII* selection gene or uninfected controls. The use of *35S:ZmKn1* resulted in a three-fold increase in shoot organogenesis relative to the *NPTII* selection. These results suggest that ZmKn1 could be used as an effective selection marker with the potential to enhance plant transformation efficiency (Luo et al. 2006). Similarly, overexpression of *ZmKn1* in transgenic citrus enhanced transformation efficiency by 3- to 15-fold (Hu et al. 2016). However, overexpression of *Nicotiana tabacum homeobox* (*NTH*) genes, *knotted1*-type homeobox genes, resulted in a range of abnormal leaf morphology. Transgenic plants overexpressing *NTH1* or *NTH9* displayed a relatively weak phenotype compared to *NTH15* or *NTH20* overexpression lines, which exhibited ectopic shoot formation on the leaf surface (Nishimura et al. 2000).

Overexpression of *BBM* enhances the spontaneous formation of somatic embryos in *Arabidopsis* and *B. napus* (Boutilier et al. 2002). *BBM* has also been used as an ectopic regulator in *T. cacao* (Florez et al. 2015) and tobacco (Srinivasan et al. 2007) genetic transformation. However, the *BBM* overexpression transgenic plants exhibited abnormal phenotypes. Thus, strategies that use inducible promoters or transgene excision to control the restricted spatiotemporal expression of *BBM* have been applied in tobacco (Srinivasan et al. 2007), *Capsicum annuum* (Heidmann et al. 2011) and *Arabidopsis* (Lutz et al. 2015). In *Populus tomentosa* genetic transformation, the generated transgenic plants are phenotypically normal when using a heat shock-inducible FRT/FLP system to excise *BBM* expression cassette from the callus stage (Deng et al. 2009). Transgenic plants generated by overexpression of *WUS* also exhibit negative pleiotropic phenotypes such as swollen hypocotyls, distorted leaves, and coiled root tips (Arroyo-Herrera et al. 2008; Bouchabke-Coussa et al. 2013; Rashid et al. 2007), suggesting that expression of *WUS* has to be strictly controlled.

A recent groundbreaking study showed that fine-tuning the expression of *WUS* and *BBM* enhanced the transformation efficiency of monocot plants (Lowe et al. 2016). Overexpression of *ZmWUS2* driven by a strong callus promoter often causes callus necrosis. To solve this problem and to induce somatic embryogenesis in immature embryos, a relatively weak *Agrobacterium*-derived nopaline synthase promoter and a strong maize Ubiquitin promoter were used to drive *ZmWUS2* (*Nos:ZmWUS2*) and *ZmBBM* (*Ubi:ZmBBM*) expression simultaneously (Lowe et al. 2016). Results showed that ectopic expression of *ZmWUS2* and *ZmBBM* significantly enhanced callus transformation efficiency in Z. *mays*, *Sorghum bicolor*, *O. sativa* and *Saccharum officinarum*. However, the continuous expression of *ZmWUS2* and *ZmBBM* leads to aberrant phenotypes, such as thick, short roots, stunted, twisted and sterile plants (Lowe et al. 2016). Thus, using desiccation-inducible promoter *rab17* to activate CRE (a recombinase enzyme isolated from the P1 bacteriophage) expression, and remove the *ZmWUS2*, *ZmBBM* and CRE expression cassettes between two loxP sites in the transformed embryogenic calli generate healthy, fertile T0 transgenic plants (Lowe et al. 2016). This strategy could also obtain transgenic plants from previously non-transformable *Z. mays* and *S. bicolor* varieties (Mookkan et al. 2017, 2018). Another strategy for solving the phenotypic abnormalities is to select suitable endogenous promoters to trigger the required spatiotemporal expression of *ZmWUS2* and *ZmBBM*. The promoter of a *Z. mays* phospholipid transferase protein gene (*ZmPLTP*) was selected to drive *ZmBBM* as *ZmPLTP* is highly expressed in leaves, embryos, and callus but has very low expression levels in roots, meristems, and reproductive tissues (Lowe et al. 2018). Somatic embryo formation was rapidly induced when *ZmPLTP:ZmBBM* and *Nos:ZmWUS2* were co-transformed into *Z. mays* immature zygotic embryos, and these somatic embryos developed into healthy fertile plants without a callus phase (Lowe et al. 2018). However, T1 seeds continuously expressing *Nos:ZmWUS2* showed poor germination. While replacing the *Nos* promoter with a *Z. mays* auxin-inducible promoter (*ZmAxig1*) and co-transformation of *ZmPLTP:ZmBBM* and *ZmAxig1:ZmWUS2* stimulated somatic embryo formation and obtained phenotypically normal transgenic plants without excision *ZmWUS2* and *ZmBBM* expression cassettes (Lowe et al. 2018). The callus-free transformation approach has been successfully tested in seven different *Z. mays* inbred lines (Lowe et al. 2018). Interestingly, a recent study showed that the *ZmPLTP:ZmWUS2* alone was sufficient to promote rapid somatic embryo formation from *Z. mays* immature embryos in a noncell autonomous manner. When transforming *Z. mays* with two *Agrobacterium* strains, one containing *ZmPLTP:ZmWUS2* and the other containing selectable and visual marker cassettes, the transformed *Z. mays* T0 plants expressed the selectable marker gene but without the integration of *ZmWUS2* (Hoerster et al. 2020). This result suggests that transformed cells expressing *ZmWUS2* could stimulate somatic embryogenesis of their neighboring cells. *ZmPLTP:ZmWUS2* also significantly shortened the tissue culture time in *S. bicolor* by inducing direct somatic embryo formation and regeneration, and also bypassed genotype-dependent callus formation (Che et al. 2022). Similarly, using two strains with one containing *ZmPLTP:ZmWUS2* and *ZmPLTP:ZmBBM* expression cassettes*,* and the other harboring a selectable marker expression cassette to transform *S. bicolor*, the transformed *S. bicolor* T0 plants expressed the selectable marker gene but without the integration of *ZmWUS2* and *ZmBBM* (Aregawi et al. 2022). This strategy increases transformation efficiency and expands amenable genotypes of different monocot species. A recent study showed that *Nos:ZmWUS2* and *3xENH–Ubi:ZmBBM* (three consecutive viral enhancers including *Figwart mosaic virus, Peanut chlorotic streak virus*, and *Mirabilis mosaic virus*) were used to improve leaf transformation efficiency and obtain plants with Cas9-mediated gene dropouts and insertion in *Z. mays* and *S. bicolor* (Wang et al. 2023). Moreover, regenerated plants were successfully obtained by using *Nos:ZmWUS2* and *3xEnh–Ubi:ZmBBM* in *Eragrostis tef*, *Panicum virgatum*, *Cenchrus americanus*, *Setaria italica*, *Secale cereale*, *Hordeum vulgare* and *O. sativa* (Wang et al. 2023). These results suggest that this may be a universal method for genetic transformation and genome editing of the Poaceae. In addition, recent studies showed that using *Nos:ZmWUS2*, *Ubi:IPT* or *Ubi:AtSTM* enhanced organogenesis in aseptic seedling leaves of *Arabidopsis*, *Nicotiana benthamiana*, and *Solanum lycopersicum*, and in mature plants of *N. benthamiana*, *Solanum tuberosum* and *Vitis vinifera* (Cody et al. 2023; Maher et al. 2020). When *Nos:ZmWUS2*, *Ubi:AtSTM* or *Ubi:IPT* were co-transformed with Cas9/gRNA plasmids, gene-edited shoots were obtained without tissue culture. The tissue culture-free method has great potential to accelerate the breeding process for many plant species (Cody et al. 2023; Maher et al. 2020).

In contrast to the adverse effects of ectopic expression of *ZmWUS2* and *ZmBBM*, overexpression of transcription factor encoding genes *Growth-regulating factor* (*GRF*) and/or its cofactor *GRF-interacting factor1* (*GIF*) does not cause aberrant phenotypes in transgenic plants. In callus induction and plant regeneration, the GRF-GIF recruits SWITCH2/SUCROSE NONFERMENTING 2 chromatin remodeling complexes to confer the meristematic potential of the proliferative tissue during organogenesis. Accordingly, overexpression of *AtGRF5* or *GRF5* orthologs enhanced transformation efficiency in *Beta vulgaris*, *B. napus*, *G. max*, *Helianthus annuusl* and *Z. mays* (Kong et al. 2020). Furthermore, a fused GRF4-GIF1 chimeric protein increases transformation efficiency and accelerates the speed of regeneration in *T. aestivum*, *O. sativa*, and citrus (Debernardi et al. 2020). Compared with the control, the transformation efficiency with the chimeric GRF4-GIF1 protein expression was increased by 7.8-, 2.1- and 4.7- fold in *T. aestivum*, *O. sativa*, and citrus, respectively (Debernardi et al. 2020). Similarly, overexpression of *GRF5,* or *GRF4* and *GIF1* also achieved high transformation efficiency in *Citrullus lanatus*. *AtGRF5,* or *ClGRF4* and *ClGIF1* factors also facilitate efficient transformation and increase CRISPR/Cas9-based genome editing efficiency in *C. lanatus* (Feng et al. 2021; Pan et al. 2022b) (Table 1).

**Table 1:** Summary of developmental regulatory genes applied to plant transformation

No.	Gene expression cassette	*Agrobacterium* strains	Transformed species	Explant	Regeneration	Regenerated plant	Transform efficiency increases	Transgene excision	Abnormal phenotype	References
1	*35S:CcSERK1*	LBA4404	*Coffea canephora*	Leaf	Embryogenesis	No	N.A.	No	No	Perez-Pascual et al. (2018)
2	*35S:AtSERK1*	C58C1	*Arabidopsis thaliana*	Flower bud	Embryogenesis	No	N.A.	No	No	Hecht et al. (2001)
3	*35S:OsSERK1*	EHA105	*Oryza sativa*	Callus	Embryogenesis	No	N.A.	No	No	Hu et al. (2005)
4	*pER8:AtESR1*	EHA105	*Arabidopsis thaliana*	Root	Organogenesis	Yes	↑	No	No	Banno et al. (2001)
5	*35S:AtPLT5*	GV3101	*Antirrhinum majus*	Mature plant	Organogenesis	Yes	↑	No	Yes	Lian et al. (2022)
	*35S:AtPLT5*	GV3101	*Brassica rapa*	Cotyledon	Organogenesis	Yes	↑	No	Yes	
6	*35S:AtCUC1*, *CUC2*	MP90	*Arabidopsis thaliana*	Callus	Organogenesis	Yes	↑	No	Yes	Daimon et al. (2003)
7	*pER10:AtESR2*	EHA105	*Arabidopsis thaliana*	Flower bud and Root	Organogenesis	Yes	↑	No	No	Ikeda et al. (2006)
8	*AtMP:AtMPΔ*	N.A	*Arabidopsis thaliana*	Root, Cotyledon, Leaf and Petiole	Organogenesis	Yes	N.A.	No	Yes	Ckurshumova et al. (2014) and Krogan et al. (2012)
9	*35S:AtLEC1*	GV3101	*Arabidopsis thaliana*	N.A	Embryogenesis	No	N.A.	No	No	Lotan et al. (1998)
10	*pER8:PaHAP3A*	C58C1	*Picea abies*	Embryogenic culture	Embryogenesis	No	N.A.	No	No	Uddenberg et al. (2016)
11	*35S:CsL1L*	EHA105	*Citrus sinensis*	Epicotyl and embryogenic callus	Embryogenesis	No	N.A.	No	No	Zhu et al. (2014)
12	*35S:TcLEC2-GR*	AGL1	*Theobroma cacao*	Cotyledon	Embryogenesis	No	N.A.	No	No	Shires et al. (2017)
13	*pER8:AtLEC22*	LBA4404	*Nicotiana tabacum*	Leaf	Organogenesis	Yes	N.A.	No	Yes	Rashid et al. (2007)
14	*35S:AtAGL15*	N.A	*Arabidopsis thaliana*	Cotyledon	Embryogenesis	No	N.A.	No	No	Harding et al. (2003)
15	*35S:GmAGL15*	–	*Glycine max*	Cotyledon	Embryogenesis	Yes	N.A.	No	Yes	Thakare et al. (2008)
16	*35S:GhAGL15s*	LBA4404	*Gossypium hirsutum*	Hypocotyl	Embryogenesis	No	N.A.	No	No	Yang et al. (2014)
17	*Ubi:TaWOX5*	C58C1	*Triticum aestivum and Zea mays*	Immature embryo	Organogenesis	Yes	↑	No	No	Wang et al. (2022a)
18	*pER8:AtWOX2/8 pER8:AtWOX2/9*	AGL1	*Nicotiana tabacum*	Leaf	Organogenesis	Yes	N.A.	No	Yes	Kyo et al. (2018)
19	*35S:ZmKn1*	LBA4404	*Nicotiana tabacum*	Leaf	Organogenesis	Yes	↑threefold	No	Yes	Luo et al. (2006)
20	*35S:ZmKn1*	EHA105	*Citrus sinensis*	Internodal stem	Organogenesis	Yes	↑3–15 fold	No	Yes	Hu et al. (2016)
21	*35S:NtNTH1, 9, 15, 20, 22*	LBA4404	*Nicotiana tabacum*	Leaf	Organogenesis	Yes	N.A.	No	Yes	Nishimura et al. (2000)
22	*35S:BnBBM UBI:BnBBM*	C58C1pMP90	*Arabidopsis thaliana*	N.A	Embryogenesis	No	N.A.	No	Yes	Boutilier et al. (2002)
	*35S:BnBBM UBI:BnBBM*	C58C1pMP90	*Brassica napus*	Haploid microspore	Embryogenesis	No	N.A.	No	Yes	
23	*35S:TcBBM*	AGL1	*Arabidopsis thaliana*	Flower bud	Embryogenesis	No	N.A.	No	Yes	Florez et al. (2015)
	*35S:TcBBM*	AGL1	*Theobroma cacao*	Cotyledon	Embryogenesis	No	N.A.	No	Yes	
24	*35S:AtBBM 35S:BnBBM*	C58C1	*Nicotiana tabacum*	Leaf	Organogenesis	Yes	N.A.	No	Yes	Srinivasan et al. (2007)
	*35S:AtBBM-GR 35S:BnBBM-GR*	C58C1	*Nicotiana tabacum*	Leaf	Organogenesis	Yes	N.A.	No	No	
25	*35S:BnBBM-GR*	GV3101	*Capsicum annuum*	Cotyledon	Embryogenesis	Yes	↑	No	Yes	Heidmann et al. (2011)
26	*35S:BBM-GR*	N.A	*Arabidopsis thaliana*	Flower bud	Organogenesis	Yes	↑	No	No	Lutz et al. (2015)
27	*AtHSP18.2:FLP-35S:BcBBM*	LBA4404	*Populus tomentosa*	Leaf	Embryogenesis	Yes	↑	FRT/FLP	No	Deng et al. (2009)
28	*pER10:AtWUS*	C58C1	*Coffea canephora*	Leaf	Embryogenesis	Yes	N.A.	No	Yes	Arroyo-Herrera et al. (2008)
29	*35S:AtWUS*	N.A	*Gossypium hirsutum*	Hypocotyl	Embryogenesis	No	N.A.	No	Yes	Bouchabke-Coussa et al. (2013)
30	*pER8:AtWUS*	LBA4404	*Nicotiana tabacum*	Leaf	Organogenesis	Yes	N.A.	No	Yes	Rashid et al. (2007)
31	*RAB17:CRE-Nos:ZmWUS2-Ubi:ZmBBM*	LBA4404	*Zea mays, Sorghum bicolor, Oryza sativa and Saccharum officinarum*	Immature embryo, Mature Seed, Leaf and Callus	Embryogenesis	Yes	↑	Cre-loxP	No	Lowe et al. (2016)
32	*RAB17:CRE-Nos:ZmWUS2-Ubi:ZmBBM*	AGL1 EHA101	*Zea mays and Sorghum bicolor*	Immature embryo	Embryogenesis	Yes	↑	Cre-loxP	No	Mookkan et al. (2018, 2017)
33	*ZmPLTP:ZmBBM-ZmAxig1:ZmWUS2*	LBA4404	*Zea mays*	Immature embryo	Embryogenesis	Yes	8.7–96%	No	No	Lowe et al. (2018)
34	*3* × *ENH-ZmPLTP:ZmWUS2-Nos:ZmCRC*	LBA4404	*Zea mays and Sorghum bicolor*	Immature embryo	Embryogenesis	Yes	↑	No	No	Che et al. (2022) and Hoerster et al. (2020)
35	*ZmPLTP:ZmWUS2-ZmPLTP:ZmBBM*	LBA4404	*Sorghum bicolor*	Immature embryo	Embryogenesis	Yes	↑	No	No	Aregawi et al. (2022)
36	*HSP17:CRE-Nos:ZmWUS2-3* × *ENH -Ubi:ZmBBM*	LBA4404	*Zea mays, Sorghum bicolor Eragrostis tef, Panicum virgatum, Cenchrus americanus, Setaria italica, Secale cereale, Hordeum vulgare and Oryza sativa*	Leaf	Embryogenesis	Yes	↑	Cre-loxP	No	Wang et al. (2023)
37	*Nos:ZmWUS2 Ubi:IPT*	N.A	*Arabidopsis thaliana, Nicotiana benthamiana and Solanum lycopersicum*	Agro transient	Organogenesis	Yes	N.A.	No	No	Maher et al. (2020)
	*Nos:ZmWUS2 Ubi:STM*	N.A	*Nicotiana benthamiana, Solanum tuberosum and Vitis vinifera*	Mature plant	Organogenesis	Yes	N.A.	No	No	
38	*2*×*35S:AtGRF5*	AGL1	*Beta vulgaris*	Leaf	Organogenesis	Yes	↑sixfold	No	No	Kong et al. (2020)
	*2*×*35S:BvGRF5-LIKE*									
	*PcUbi4-2:BnGRF5-LIKE*	SHA001	*Brassica napus*	Hypocotyl	Organogenesis	Yes	↑	No	No	
	*PcUbi4-2:GmGRF5-LIKE*	SHA017	*Glycine max*	Primary node	Organogenesis	Yes	↑	No	No	
	*35S:AtGRF5*	EHA105	*Helianthus annuusl*	Cotyledon	Organogenesis	Yes	↑	No	No	
	*35S:HaGRF5-LIKE*									
	*BdEF1:AtGRF5*	LBA4404	*Zea mays*	Immature embryo	Organogenesis	Yes	↑	No	No	
	*BdEF1:ZmGRF5-LIKE1*									
	*BdEF1:ZmGRF5-LIKE2*									
39	*ZmUbi:GRF4-GIF1*	EHA105	*Triticum aestivum*	Immature embryo	Organogenesis	Yes	↑7.8-fold	No	No	Debernardi et al. (2020)
	*ZmUbi:GRF4-GIF1*	EHA105	*Oryza sativa*	Callus	Organogenesis	Yes	↑2.1-fold	No	No	
	*35S:GRF4-GIF1*	EHA105	*Citrus sinensis*	Etiolated Epicotyl	Organogenesis	Yes	↑4.7-fold	No	No	
40	*35S:GRF4-GIF1*	EHA105	*Citrullus lanatus*	Cotyledon	Organogenesis	Yes	↑ninefold	No	No	Feng et al. (2021)
41	*UBQ10:AtGRF5*	GV3101	*Citrullus lanatus*	Cotyledon	Organogenesis	Yes	↑40-fold	No	No	Pan et al. (2022b)

*N.A.*, not applicable; “↑” represent increased transform efficiency

## Nanoparticle uptake and translocation in plant cells

*Agrobacterium*-mediated method is the most frequently used tool for gene delivery in plant transformation. However, this method usually requires regeneration from tissue culture and infects only some plant species. Furthermore, it is hard to use *Agrobacterium* for chloroplast or mitochondrion transformation. Nanoparticles (NPs), natural or manufactured ultradisperse objects ranging from 1 to 100 nm, are promising materials for exogenous biomolecule delivery because of their ability to traverse the plant cell without external force and their broad host applicability. The application of nanotechnology to plant cells requires understanding the interaction between NPs and plant cells, including the uptake and translocation of NPs.

### Nanoparticle uptake in plant cells

**Fig. 5:**
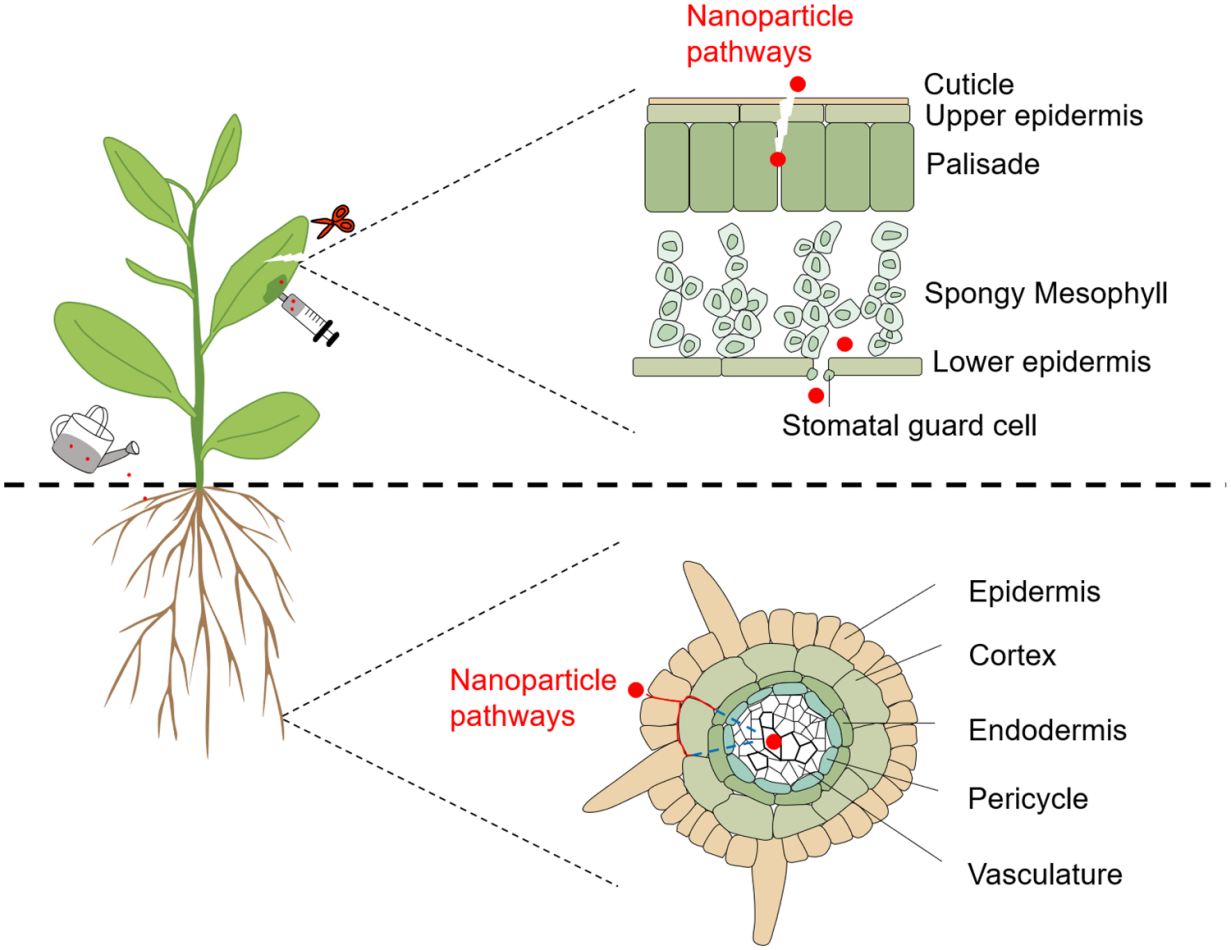
Schematic representation of uptake and translocation of nanoparticles (NPs) in plants. NPs can be applied to roots and leaves and uptaken into plants through damage or natural openings, such as stomata pores. Apoplastic and symplastic paths are the mobilization pathways of NPs after penetrating the outer protective layer of plants. Red solid lines indicate apoplastic paths, and blue dotted lines indicate symplastic paths (color figure online)

In plant science, NPs can be applied to roots and above-ground plant tissues especially leaves. Shoot surfaces are usually covered with a cuticle, which acts as a lipophilic barrier to protect primary organs of above-ground plants. NPs can enter the cell wall through natural openings, such as stomata pores (Eichert et al. 2008). Damages and wounds may also be feasible pathways for NP internalization in both aerial and hypogeal parts of plants (Al-Salim et al. 2011) (Fig. 5). In addition, delivery methods affect NP uptake efficiency in plants. A recent study showed that compared with the NP drop-cast method, the aerosol application help to improve NP uptake in *C. lanatus* (Raliya et al. 2016).

### Nanoparticle translocation in plant cells

Once penetrate the outer protective layers of plants, mobilization of NPs in plants through apoplastic and symplastic paths. Apoplastic transport takes place outside the plasma membrane through the cell wall and extracellular spaces, while symplastic transport occurs between the cytoplasm of adjacent cells connected by plasmodesmata and sieve plate pores with the movement of water and solutes. Apoplastic transport has been demonstrated to facilitate the radial movement of NPs (Gonzalez-Melendi et al. 2008; Larue et al. 2014; Sun et al. 2014). However, the longitudinal Casparian strip composed of lignin-like structures prevents this radial movement in the root endodermis (Lv et al. 2015; Sun et al. 2014), and the symplastic path could bypass this barrier (Schwab et al. 2016) (Fig. 5). The cell wall is a multi-layered structure of pore diameter ranging from 5 to 20 nm (Fleischer et al. 1999; Fujino and Itoh 1998; Zemke-White et al. 2000). Recent studies demonstrated that different types of NPs with a mean diameter between 3 and 50 nm could easily pass through *Arabidopsis* and citrus cell walls (Etxeberria et al. 2016; Torney et al. 2007). When NPs penetrate the cell wall and reach the plasma membrane, they can enter cells through endocytosis. In addition, NPs can also cross the plasma membrane directly (Chang et al. 2013). Once NPs enter the cytoplasm, plasmodesmata promote the cell-to-cell movement of NPs. The transport of NPs of various sizes through plasmodesmata has been demonstrated in some plant species (Geisler-Lee et al. 2013; Lin et al. 2009; Zhai et al. 2014).

## Application of nanoparticles in plant transformation

Over the past decade, NPs have been applied for plant delivery. Early studies showed NP-mediated plasmid DNA and protein delivery into plant cells with external force. For instance: (1) Gold-functionalized mesoporous silica nanoparticles (Au-MSNs)-mediated delivery of DNA (Torney et al. 2007) and proteins (Martin-Ortigosa et al. 2012, 2014) through the biolistic method. (2) Combined utilization of polyethylene glycol (PEG)-mediated transformation and polymeric dimethylaminoethyl metacrylate (DMAEM)-based polymers effectively deliver plasmid DNA into *Ceratodon purpureus* protoplasts and obtain stable transformants (Finiuk et al. 2017). (3) Polyethylenimine (PEI) nanoparticles deliver DNA into suspended cells of *Crocus sativus* through ultrasound, resulting in improved transfection efficiency (Firoozi et al. 2018). (4) Combined peptide-displaying micelle complexes (MCs) and cell wall-loosening zwitterionic liquid (ZIL) carry DNA into specific plant organelles through the vacuum/compression method (Miyamoto et al. 2022). Of these methods, cell-penetrating peptide-displaying MCs (CPP-MCs) was used to deliver DNA into nuclei while combined CPP-MCs and chloroplast-targeting peptide-displaying MCs (CPP/CTP-MCs) could be used for delivering DNA into chloroplasts (Table 2).

**Table 2:** Summary of nanoparticles applied to plant transformation

No.	Nanoparticles	Function modification	Delivery method	Cargo type	Target species/tissue	Expression	References
1	Mesoporous silica nanoparticles	Gold nanoparticles	Particle bombardment	Plasmid DNA and Chemicals	*Nicotiana tabacum*/Cotyledon and *Zea mays*/Immature embryo	Transient and stable expression	Torney et al. (2007)
2	Mesoporous silica nanoparticles	Gold nanoparticles	Particle bombardment	Protein	*Zea mays*/Immature embryo	Transient and stable expression	Martin-Ortigosa et al. (2014)
3	Mesoporous silica nanoparticles	Gold nanoparticles	Particle bombardment	Plasmid DNA and Protein	*Nicotiana tabacum* and *Euchlaena mexicana*/Leaf	Transient expression	Martin-Ortigosa et al. (2012)
4	Polymeric dimethylaminoethyl metacrylate-based polymers	–	Incubation and PEG-mediated transformation	Plasmid DNA	*Nicotiana tabacum and Ceratodon purpureus*/Protoplast	Transient and stable expression	Finiuk et al. (2017)
5	PEI nanoparticles	–	Ultrasound	DNA	*Crocus sativus*/Suspended cell	Transient expression	Firoozi et al. (2018)
6	Micelle complexes	Cell-penetrating peptide and chloroplast-targeting peptide	Vacuum/compression method	Plasmid DNA	*Arabidopsis thaliana*/Seedling and Leaf	Transient expression	Miyamoto et al. (2022)
7	Mesoporous silica nanoparticles	TMAPS, APTMS, THPMP	Infiltration	Plasmid DNA	*Arabidopsis thaliana* /Root	Transient expression	Chang et al. (2013)
8	Single-walled carbon nanotubes	Polyethylenimine	Infiltration and incubation	Plasmid DNA	*Nicotiana benthamiana*, *Eruca sativa*, *Triticum aestivum* and *Gossypium hirsutum*/Leaf and *Eruca sativa*/Protoplast	Transient expression	Demirer et al. (2019a, b)
9	Single-walled carbon nanotubes	Super-purified HiPCO SWNTs	Infiltration	Small interfering RNA	*Nicotiana benthamiana*/Leaf	Transient expression	Demirer et al. (2020)
10	Single-walled carbon nanotubes	Chitosan	Infiltration and incubation	Plasmid DNA	*Eruca sativa*, *Nasturtium officinale*, *Spinacia oleracea* and *Nicotiana tabacum*/Leaf and *Arabidopsis thaliana*/Protoplast	Transient expression	Kwak et al. (2019)
11	Carbon dots	Polyethylenimine	Spray	Small interfering RNA	*Nicotiana benthamiana* and *Solanum lycopersicum*/Leaf	Transient expression	Schwartz et al. (2020)
12	Carbon dots	Polyethylenimine	Infiltration	Plasmid DNA	*Oryza Sativa*, *Triticum aestivum* and *Phaseolus radiatus*/Leaf and *Oryza Sativa*/Root	Transient expression	Wang et al. (2020)
13	Gold nanoclusters	Polyethylenimine	Infiltration	Small interfering RNA	*Nicotiana benthamiana*/Leaf	Transient expression	Zhang et al. (2021a)
14	DNA nanostructures	–	Infiltration	Small interfering RNA	*Nicotiana benthamiana*/Leaf	Transient expression	Zhang et al. (2019)
15	Layered double hydroxide nanoparticles	–	Injection	Double-stranded RNA	*Solanum lycopersicum*/Flower pedicel	Transient expression	Molesini et al. (2022)
16	Graphene oxide nanoparticles	Polyethylenimine and polyethylene glycol	Infiltration	Small interfering RNA	*Nicotiana benthamiana*/Leaf	Transient expression	Li et al. (2022)
17	Chitosan nanoparticles	–	Incubation	Plasmid DNA	*Paulownia tomentosa*/Nodal segment	Stable expression	Hussien (2020)
18	Chitosan nanoparticles	–	Inoculation	Plasmid DNA	*Allium cepa*/Seedling	Stable expression	Hussien et al. (2022)
19	Magnetic nanoparticles	Polyethylenimine	Magnetic field	Plasmid DNA	*Gossypium hirsutum*, *Capsicum annuum*, *Cucurbita moschata*, *Cucurbita pepo* and *Lilium brownii*/Pollen	Stable expression	Zhao et al. (2017)
20	Magnetic nanoparticles	–	Magnetic field	Plasmid DNA	*Zea mays*/Pollen	Stable expression	Wang et al. (2022b)

**Fig. 6:**
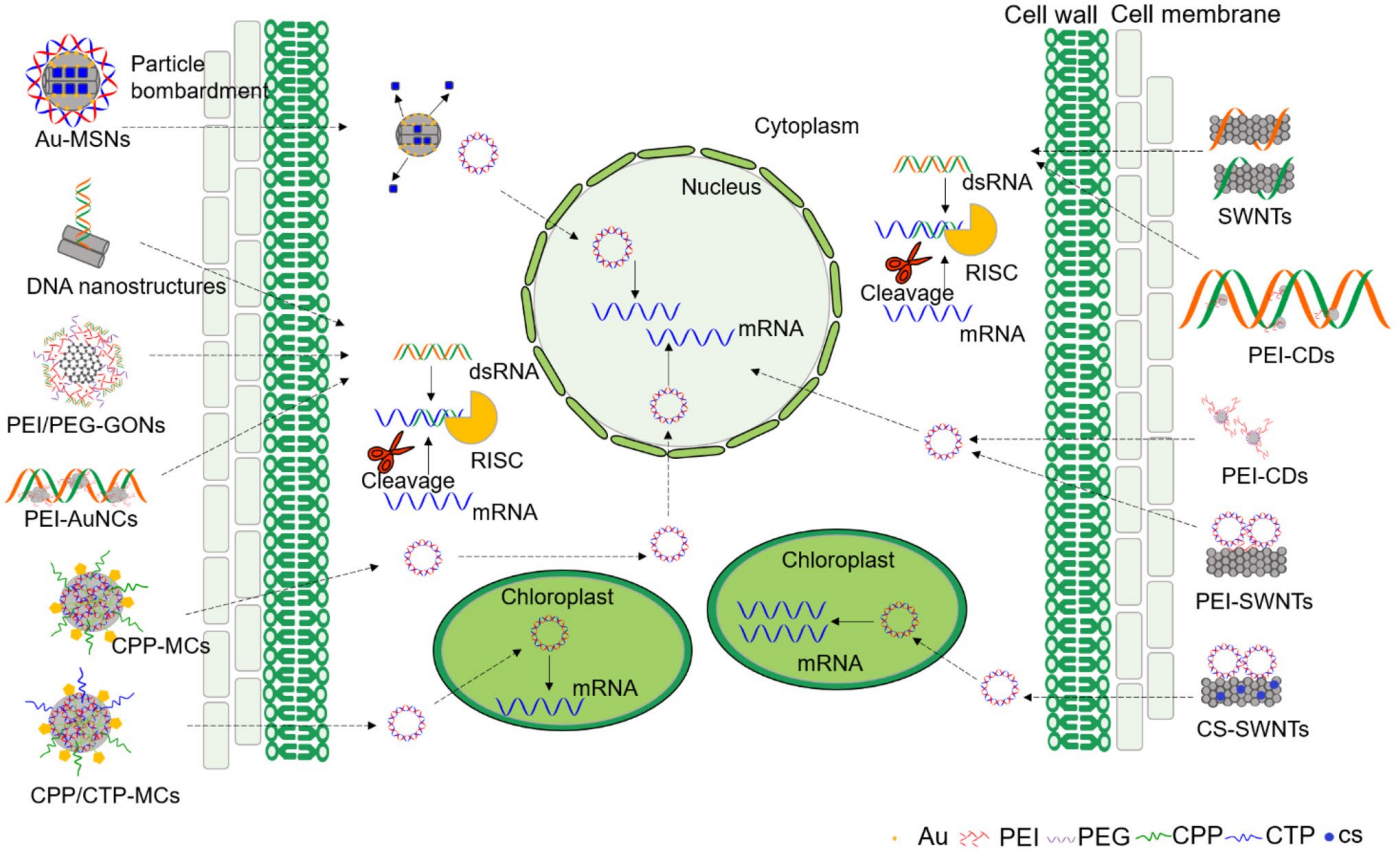
Schematic illustration of nanoparticle (NP) structures and NP-mediated nucleic acid and protein delivery into plant leaf cells. *Au-MSNs* gold-functionalized mesoporous silica nanoparticles, *CPP-MCs* cell-penetrating peptide-displaying micelle complexes, *CPP/CTP-MCs* combined CPP-MCs and chloroplast-targeting peptide-displaying MCs, *CS-SWNTs* chitosan-complexed single-walled carbon nanotubes, *PEI-AuNCs* Polyethylenimine-functionalized gold nanoclusters, *PEI-CDs* PEI-functionalized carbon dots, *PEI/PEG-GONs* PEI/polyethylene glycol (polymer)-functionalized graphene oxide nanoparticles, *PEI-SWNTs* PEI-functionalized single-walled carbon nanotubes, *RISC* RNA-induced silencing complex, *SWNTs* single-walled carbon nanotubes

However, other studies have demonstrated that NPs can pass through plant cell walls without external force: (1) MSNs-mediated foreign DNA delivery into intact *Arabidopsis* roots without mechanical force (Chang et al. 2013). (2) Application of single-walled carbon nanotubes (SWNTs) for the delivery of small interfering RNA (siRNA) and plasmid DNA into a variety of plant species (Demirer et al. 2020, 2019a, b; Kwak et al. 2019). In addition, chitosan-complexed single-walled carbon nanotubes (CS-SWNTs) could deliver plasmid DNA into chloroplasts of mature *Eruca sativa*, *Nasturtium officinale*, *Spinacia oleracea*, tobacco plants and isolated *Arabidopsis* mesophyll protoplasts (Kwak et al. 2019). (3) Using PEI functionalized carbon dots (CDs) to efficiently deliver plasmid DNA or siRNA into intact plants (Schwartz et al. 2020; Wang et al. 2020). (4) DNA nanostructures and PEI functionalized gold nanoclusters (PEI-AuNCs) internalize into plant mature cells and deliver a siRNA to silence green fluorescent protein (GFP) expression in transgenic *N. benthamiana* plants (Zhang et al. 2021a; Zhang et al. 2019). Moreover, a recent study has reported that the double-stranded RNA (dsRNA) can be coupled to layered double hydroxides (LDH) nanoparticles, inducing gene silencing through injection into *S. lycopersicum* flower pedicel (Molesini et al. 2022). Another recent study used polymer-functionalized graphene oxide nanoparticles (GONs) to deliver siRNAs into intact *N. benthamiana* cells (Li et al. 2022). These successful applications indicate that NPs have great potential for plant delivery (Fig. 6, Table 2).

To fully leverage NPs for plant genetic engineering, it is essential to achieve stable transformation enabling the generation of transgenic plants. Chitosan nanoparticles can deliver a thionin gene with antimicrobial properties into *Allium cepa* and *Paulownia tomentosa* cells, producing transgenic *A. cepa* and *P. tomentosa* resistance to black rot diseases and bacterial infection, respectively (Hussien 2020; Hussien et al. 2022). A groundbreaking study showed that stable genetic transformation had been successfully achieved in *G. hirsutum* plants using magnetic nanoparticles (MNPs) technology (Zhao et al. 2017). In this system, the BTΔα-CPTI gene-MNPs complex is delivered into *G. hirsutum* pollen under a magnetic field. Pollen magnetofection not only perfectly protects foreign DNA integrity, but also maintains pollen viability. Insect-resistant transformed plants are successfully generated through magnetofected pollen pollination. The exogenous gene was successfully integrated into the genome, effectively transcribed, and stably inherited into the offspring (Zhao et al. 2017). Y18 and SU12, two previously difficult-to-transform *G. hirsutum* varieties, are successfully transformed using this system. In addition, genetically modified *C. annuum* and *Cucurbita moschata* plants have also been successfully created (Zhao et al. 2017). A recent study reported that the MNPs system was also used to deliver exogenous genes to different *Z. mays* inbred lines and successfully obtained transgenic plants (Wang et al. 2022b). Further investigation found that transfection with a cool temperature pretreatment of pollen to open the germination aperture can improve the efficiency of DNA entry and maintain pollen viability (Wang et al. 2022b). As this method is genotype-independent, culture-free, and easy to handle, it has great potential to transform recalcitrant and genotype-dependent crops and thus accelerate the breeding process (Table 2).

## Conclusions and perspectives

Many developmental regulatory genes have been shown to work effectively both in dicots and monocots (Table 1), and manipulation of these genes has great potential for developing genotype-independent genetic transformation methods in various crops. However, constitutive expression of developmental regulatory genes, such as *ZmWUS2* and *ZmBBM* often interferes with normal plant development and leads to negative pleiotropic effects. So fine-tuning the expression of these genes is essential for applying them to plant transformation (Hoerster et al. 2020; Lowe et al. 2016, 2018; Mookkan et al. 2018, 2017). Overexpression of *GRF* and/or *GIF* can improve the transformation efficiency in a variety of crops but did not cause abnormal phenotypes in transgenic plants (Debernardi et al. 2020; Kong et al. 2020). This may be due to the post-transcriptional down-regulation of *GRF* by endougenous *miRNA396* in T0 plants, which provides a built-in mechanism for alleviating pleiotropic problems (Debernardi et al. 2020; Li et al. 2021). There are many genes, such as ABI3 and *LBDs*, affecting plant regeneration have not been used for plant transformation. It is worth investigating whether fine-tuning the expression of these genes could facilitate the improvement of plant transformation. A recent study has established a versatile CRISPR-Combo platform for simultaneous genome editing and gene activation in plants (Pan et al. 2022a). This system can be applied to achieve plant regeneration by simultaneously activating *BBM1* and editing the genome at *Grain weight2* (*GW2*) and *Grain number 1a* (*GN1a*) loci without exogenous hormone application in *O. sativa* (Pan et al. 2022a). This system has promising application prospects in crop breeding.

Over the past decade, NPs have been widely used to deliver genes and proteins into plant cells (Demirer et al. 2019a; Kwak et al. 2019; Martin-Ortigosa et al. 2014). However, most of them are transient transformations of foreign genes. To fully leverage NPs for plant genetic engineering, transgenes have to be stably inherited to the next generation. Currently, MNPs have successfully achieved stable genetic transformation in *G. hirsutum*, *C. annuum*, *C. moschata*, and *Z. mays* (Wang et al. 2022b; Zhao et al. 2017). However, a recent study reported that the transfection of *Lilium brownii*, *S. bicolor*, and *Z. mays* pollens by MNPs was unsuccessful (Vejlupkova et al. 2020), possibly due to the structure of the single aperture on the pollen wall and the entry of exogenous DNA is blocked when the aperture is covered by wall material or the operculum. Indeed, promoting aperture open by pretreating maize pollens at cool temperatures facilitates exogenous DNA entry and expression (Wang et al. 2022b), which opens a window for applying NP-mediated plant transformation in troublesome plant species. Overall, either manipulation of developmental regulatory genes or nanotechnology facilitates genotype-independent plant genetic transformation and further promotes functional genome research and crop breeding.

## Data Availability

This manuscript is a review, no data was used for the research described in the article.

## Abbreviations

ABI: Abscisic acid insensitive
AGL: Agamous-like
ARF: Auxin response factor
ARR: Arabidopsis response regulator
ATP: Adenosine triphosphate
ATXR: Arabidopsis trithorax-related
Au-MSN: Gold-functionalized mesoporous silica nanoparticle
AuNC: Gold nanocluster
AuxRE: Auxin response element
BBM: Baby boom
CD: Carbon dot
CIM: Callus-inducing medium
CPP: Cell-penetrating peptide
CTP: Chloroplast-targeting peptide
CUC: Cup-shaped cotyledon
CYCD: Cyclin D
DMAEM: Dimethylaminoethyl metacrylate
DRN: Dornröschen
dsRNA: Double-stranded RNA
ESR: Enhancer of shoot regeneration
FUS: FUSCA
GFP: Green fluorescent protein
GIF: GRF-interacting factor
GN: Grain number
GON: Graphene oxide nanoparticle
GRF: Growth-regulating factor
GW: Grain weight
HDAC: Histone deacetylases
H3K27me3: Histone H3 lysine 27 trimethylation
H3K36me3: Histone H3 lysine 36 trimethylation
iPB: *in planta* Particle bombardment
IPT: Isopentenyl transferase
JMJ: JUMONJI C domain-containing protein
LBD: Lateral organ boundaries domain
LDH: Layered double hydroxide
LEC: Leafy cotyledon
LRR: Leu-rich repeat
MC: Micelle complex
MET: Methyltransferase
MNP: Magnetic nanoparticle
MP: Monopteros
NM: Nanomaterial
NP: Nanoparticle
NTH: *Nicotiana tabacum* Homeobox
PEG: Polyethylene glycol
PEI: Polyethylenimine
PIN: PIN-PORMED
PLK: Receptor-like kinase
PLT: PLETHORA
PRC: POLYCOMB REPRESSIVE COMPLEX
RIM: Root-inducing medium
SAM: Shoot apical meristem
SERK: Somatic embryogenesis receptor-like kinase
SIM: Shoot-inducing medium
siRNA: Small interfering RNA
STM: Shoot meristemless
SWNT: Single-walled carbon nanotube
TSA: Trichostatin A
Vir: Virulence
WIND: Wound-induced dedifferentiation
WOX: Wuschel related homeobox
WUS: WUSCHEL
YUC: YUCCA
ZIL: Zwitterionic liquid
